# Recurrent Hypoglycaemia Leading to Early Diagnosis of Septo‐Optic Dysplasia in a Small‐for‐Gestational‐Age Infant—A Case Report

**DOI:** 10.1002/ccr3.71985

**Published:** 2026-02-09

**Authors:** Yuan Rui Leon Tan, Xiaoao Dong, Ngee Lek, Suresh Chandran, Odattil Geetha

**Affiliations:** ^1^ Department of Neonatology KK Women's and Children's Hospital Singapore; ^2^ Department of Pediatrics KK Women's and Children's Hospital Singapore; ^3^ Paediatrics Academic Clinical Program Lee Kong Chian School of Medicine Singapore; ^4^ Paediatrics Academic Clinical Program Duke‐NUS Medical School Singapore; ^5^ Paediatrics Academic Clinical Program Yong Loo Lin School of Medicine Singapore

**Keywords:** case report, cholestasis, hypopituitarism, neonate, recurrent hypoglycaemia, septo‐optic dysplasia

## Abstract

Septo‐optic dysplasia (SOD) is a rare condition with highly heterogenous clinical manifestations and can be a diagnostic challenge. It can present with pituitary hormone deficiencies, growth failure, visual impairment, and neurological symptoms. SOD can be diagnosed at different time points—from the prenatal period to childhood. Our team cared for a baby girl who presented with recurrent hypoglycaemia, conjugated hyperbilirubinemia, and hormonal deficiencies, prompting investigations that resulted in an early diagnosis of SOD. This case report highlights the importance of considering neuroimaging to exclude septo‐optic dysplasia in term infants with recurrent hypoglycaemia, low cortisol, and growth hormone levels, as timely diagnosis with early hormone replacement reduces long‐term morbidities.

**Key Clinical Message**

It is important to consider neuroimaging to exclude septo‐optic dysplasia in term infants with recurrent hypoglycaemia, low cortisol and growth hormone levels, as timely diagnosis with early hormone replacement reduces long term morbidities.

## 1 Introduction

Septo‐optic dysplasia (SOD), i.e., de Morsier Syndrome, is a congenital syndrome characterized by two‐or‐more features of the classical triad: (i) optic nerve dysplasia, (ii) hypothalamic–pituitary axis dysfunction, and (iii) midline brain defect [1, 2, 3]. SOD is rare, with an incidence of 1 in 10,000 live births [4]. This heterogenous disorder can have high developmental phenotypic variability and additional systemic anomalies. They can present at varying time points—from birth with congenital abnormalities to the neonatal period with symptoms of pituitary hormone deficiencies or later in childhood with growth failure, visual impairment, and neurological manifestations [4]. This is supported with a case series by Nalawade et al. who reported a broad diagnostic range with median diagnosis age of 5 years [5], and another case series by Al‐Senawi et al. who reported patients presenting between 3 months and 1 year of age [6]. Early diagnosis and treatment entail better outcomes, reducing the risk of adrenal crisis and hypoglycaemia and allowing optimal growth and development. However, diagnosis in the neonatal period based on metabolic or endocrine presentation is uncommon, making this case particularly instructive.

We report a small‐for‐gestational‐age (SGA) infant presenting in the neonatal period with recurrent hypoglycaemia, hypothermia, and cholestasis, with an eventual diagnosis of congenital hypopituitarism.

## 2 Case History and Examination

A female asymmetrically SGA infant weighing 2696 g (3rd percentile, −1.61 SDS) with a head circumference of 33 cm (13th percentile, −1.15 SDS) and length of 46.5 cm (5th percentile, −1.58 SDS) was born to a young primigravida mother at 40+ 1 week, with an Apgar score of 9 at 1 and 5 min of life. Fetal scans did not reveal any stigmata related to SOD. The infant's physical examination was unremarkable. There is no family history of endocrine disorders, and the parents are non‐consanguineous. The mother mainly followed a traditional Chinese style diet common in our local population, without any special supplement use. Maternal serologies were unremarkable. The parents are both young healthy adults with strong family support. The mother denied any smoking.

**FIGURE 1:**
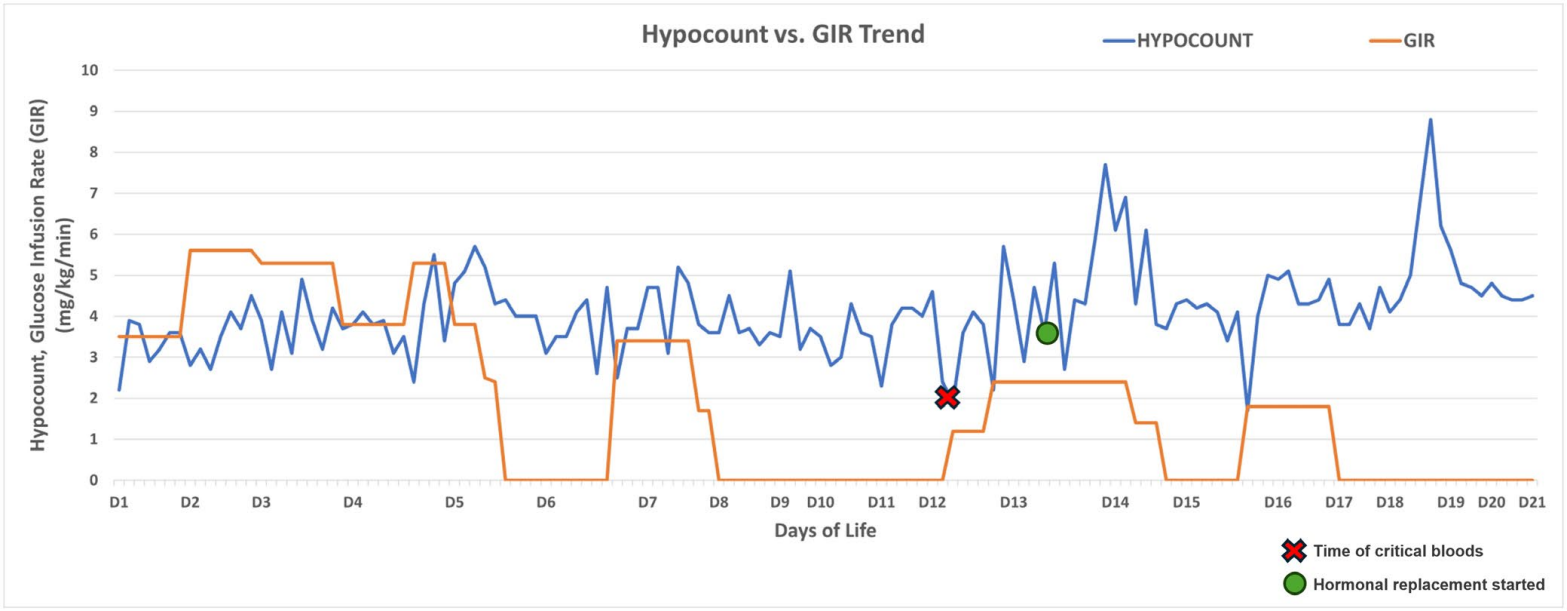
Blood glucose and glucose infusion rate (GIR) trend in our child.

She was an SGA infant at risk of glucose dysregulation and was enrolled in the hypoglycaemia screening program, which involves regular capillary blood glucose monitoring in the first 24–36 h of life [7]. At 9 h of life, she developed symptomatic hypoglycaemia (capillary blood glucose (CBG), 2.2 mmol/L) with hypothermia (34.9°C) and was lethargic. Following our departmental guidelines, she was given milk feeds after the buccal glucose gel application [7, 8]. Hypoglycaemia persisted, and she was commenced on intravenous glucose infusion. She was treated with antibiotics for concerns of neonatal sepsis, which was discontinued after 48 h with normal septic markers and negative cultures. She required a glucose infusion rate (GIR) of 5.5 mg/kg/min and a feed volume of 189 mL/kg/day by day 10 of life. However, she had recurrent episodes of hypoglycaemia (defined as capillary glucose < 3.0 mmol/L) [7] with a glucose nadir of 1.6 mmol/L on attempting to wean off the GIR (Figure 1), with occasional hypothermic episodes.

## 3 Differential Diagnosis, Investigations and Treatment

Critical blood was done on day 12 of life during an episode of hypoglycaemia while on full feeds to rule out hyperinsulinism, but insulin was undetectable (Table 1). Low cortisol and growth hormone (GH) prompted further investigations (Table 2). Biochemical evidence of multiple pituitary hormone deficiencies, including hypocortisolism, central hypothyroidism, and GH deficiency with probable gonadotropin deficiencies, was noted. Whilst on monitoring, the baby was also noted to have hyponatremia (Na 126 mmol/L) with normal serum potassium (K 4.7 mmol/L) and normal renal function, but hyponatremia self‐corrected to Na 133 mmol/L within 24 h without sodium supplements.

**TABLE 1:** Results of critical blood for hypoglycemia done on day 12 of life.

Test	Results	Reference range
Plasma glucose	1.6 mmol/L	3.0–6.9 mmol/L
Cortisol	< 28 nmol/L	> 350 nmol/L
Growth hormone	1.4 μg/L	0–8 μg/L
Insulin	Undetectable	< 1.6 mU/L
Ketones	0.0 mmol/L	≥ 0.6 mmol/L

**TABLE 2:** Thyroid function test, sex hormones and renal panel done on day 12 of life.

Test	Results	Reference range
Thyroid stimulating hormone	4.54 mIU/L	0.50–6.50 mIU/L
Free thyroxine (T4)	6.9 pmol/L	11.6–28.3 pmol/L
Estradiol (E2)	< 88 pmol/L	< 2382 pmol/L
Luteinizing Hormone (LH)	0.2 IU/L	0.6–89.1 IU/L
Follicle stimulation hormone (FSH)	1.8 IU/L	1.4–16.7 IU/L
Progesterone	1.9 nmol/L	≤ 50.6 nmol/L
Serum sodium	126 mmol/L	133–146 mmol/L
Serum potassium	4.7 mmol/L	3.7–5.9 mmol/L
Serum chloride	97 mmol/L	98–113 mmol/L
Serum bicarbonate	20 mmol/L	14–22 mmol/L
Serum creatinine	29 μmol/L	29–82 μmol/L
Serum urea	1.8 mmol/L	1.0–8.2 mmol/L

On day 9 of life, she developed clay‐colored stools, and the liver function test (LFT) revealed conjugated hyperbilirubinemia without transaminitis (Table 3), with conjugated hyperbilirubinemia defined as direct bilirubin levels of ≥ 20 μmol/L.

**TABLE 3:** Liver function test done on day 9 of life.

Test (serum)	Results	Reference range
Albumin	30 g/L	28–41 g/L
Gamma‐glutamyl transferase	528 U/L	21–201 U/L
Aspartate transaminase	29 U/L	32–163 U/L
Bilirubin direct	25 μmol/L	6–12 μmol/L
Alanine transaminase	12 U/L	5–33 U/L
Alkaline phosphatase	229 U/L	96–291 U/L
Total protein	55 g/L	53–83 g/L
Total bilirubin	242 μmol/L	0–100 μmol/L

**FIGURE 2:**
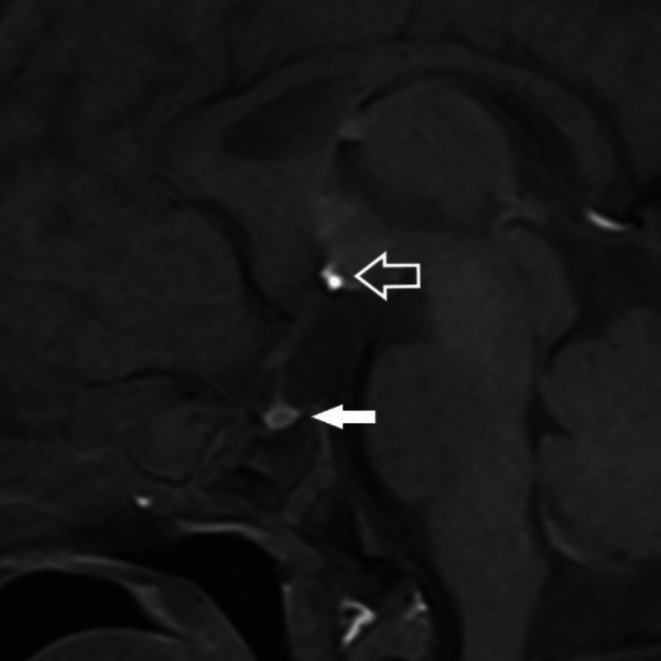
Sagittal T1‐weighted MR image of the pituitary gland shows the adenohypophysis situated in the pituitary fossa (solid arrow). The neurohypophysis is not situated in the pituitary fossa but ectopically situated in the floor of the third ventricle‐hypothalamus (open arrow).

**FIGURE 3:**
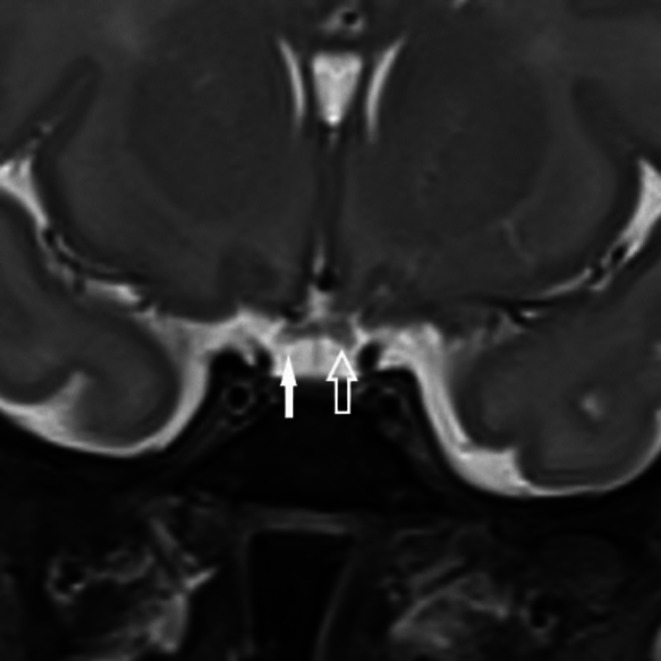
Coronal T2‐weighted fine section MR image shows the right aspect of the optic chiasm (solid arrow) to be slightly smaller than the left (open arrow).

Cranial ultrasound scan on day 13 of life was unremarkable. Magnetic Resonance Imaging (MRI) of the brain revealed ectopic neurohypophysis in the floor of the 3rd ventricle floor‐hypothalamus and adenohypophysis in the pituitary fossa (Figure 2). The orbit images showed hypoplasia of the right optic nerve and chiasm (Figure 3). Fundoscopic examination revealed bilateral optic disc hypoplasia.

Further work‐up for other differential diagnoses of neonatal cholestasis was done on days 9–10 of life. Intrahepatic causes such as cytomegalovirus infection were investigated and returned negative. A hepatobiliary system scan was done and returned normal. Urinalysis performed returned unremarkable. Genetic testing for other causes of conjugated hyperbilirubinemia was not performed. Cholestasis was conservatively managed, with increased stool pigmentation and normalization of LFT following correction of pituitary insufficiency, suggesting that cholestasis was secondary to hypopituitarism.

## 4 Outcome and Follow‐Up

A diagnosis of SOD was made. Glucocorticoid and levothyroxine replacement was initiated on day 13 of life. Resolution of hypoglycaemia was noted within 2 days, with only one further episode of hypoglycemia on day 14 of life, and nil subsequent hypoglycemic episodes thereafter. Her thyroxine (T4) normalized in 4 days. Parents were educated on stress dose adjustments during illness, and given written instructions to increase hydrocortisone during sick days. She was discharged home on day 21 of life weighing 2645 g (< 3rd percentile, −3.1 SDS).

She was followed up by a team comprising neonatologists, endocrinologists, gastroenterologists, ophthalmologists, and early intervention therapists. On day 46 of life, her weight was 3700 g (< 3rd percentile, −1.54 SDS), but weight gain was 28 g/day over the last 30 days. On follow‐up at 1 year 7 months of age, she weighed 8700 g (3rd to 10th centile, −1.5 SDS) and measured 70 cm (< 3rd centile, −3.5 SDS), and is able to walk independently and call “papa” and “mama”.

She is on follow up with the ophthalmologist, with parents reporting that she is able to play and navigate her environment without any overt visual difficulty. Formal visual assessments like retinoscopy are challenging at this age. The ophthalmologist has advised for continued visual development monitoring.

She is on hydrocortisone, levothyroxine replacement and recombinant human growth hormone (rhGH). She is on regular pediatric endocrine outpatient reviews to review her growth and optimize the doses of medications. Her thyroid function tests are repeated every 3 months on follow up visits.

## 5 Discussion

Septo‐optic dysplasia is diagnosed when there are at least two or more features of the classical triad: (i) optic nerve dysplasia, (ii) hypothalamic–pituitary axis dysfunction, and (iii) midline brain abnormalities (e.g., absence of the septum pellucidum or agenesis of the corpus callosum, and hypoplasia of the optic chiasma/nerves) [1, 2, 3, 9].

Patients with SOD have highly heterogenous clinical manifestations—with only about 30% of SOD cases having the complete triad and 62% having endocrinopathies from hypothalamic–pituitary dysfunction [10]. SOD can be suspected during a fetal scan with the absence of cavum septum pellucidum and abnormally detected optic nerves, which were normal in our fetal imaging [11]. In a case series by Nalawade et al., 50% had isolated absent septum pellucidum, 30% had isolated corpus callosum agenesis, whilst 15% had both absent septum pellucidum and agenesis of the corpus callosum [5]. Garcia‐Filion et al. cited that visual manifestations represent 60%–90% of presentations to physicians in patients with SOD [12]. This finding was supported by the case series by Ferran et al., in which the diagnosis of optic nerve hypoplasia preceded the diagnosis of SOD in all 5 cases [13]. Our case underscores the value of a robust early hypoglycemia screening programme, which led to the identification of multiple pituitary hormone deficiencies, with MRI subsequently confirming ectopic neurohypophysis and optic nerve hypoplasia.

The onset and extent of endocrinopathies in patients with SOD can vary considerably. In a Japanese case series by Koizumi et al., seizures, developmental delay, and cerebral palsy are the most frequent neurological associations noted. Other SOD‐associated features include sleep disturbance, precocious puberty, obesity, anosmia, sensorineural hearing loss, and cardiac anomalies. Diabetes insipidus and hypogonadotropic hypogonadism are also reported [14]. In these cases, it is recommended to do further blood investigations to evaluate the levels of GH, cortisol, gonadotropin, serum prolactin, thyroid stimulating hormone (TSH), adrenocorticotropic hormone (ACTH), and testosterone. Growth hormone deficiency is the most common pituitary hormone deficiency, followed by deficiencies of TSH and ACTH. In rare cases, diabetes insipidus was reported [15, 16]. Children with GH deficiency were traditionally thought to present in early childhood with short stature. They may present with neonatal hypoglycaemia, as GH is also one of the counter‐regulatory hormones involved in gluconeogenesis during fasting. Patients with multiple pituitary hormone deficiencies have been found to have a greater incidence of hypoglycaemia than those with isolated GH deficiency [17]. In our case, being an SGA infant, poor glycogen stores were initially considered as the cause of her recurrent hypoglycaemia, with other differentials including hyperinsulinism and adrenal insufficiency. Critical blood played a crucial role in the workup of our case, as it showed appropriate insulin response to low glucose with low ketones due to poor muscle mass and decreased fat stores in SGA infants. Low cortisol level pointed towards pituitary insufficiency in the absence of hyperinsulinism, prompting the differential diagnosis of SOD as early as day 13 of life, given cortisol's role in fuel mobilization [18].

Pituitary hormone deficiencies represent a rare cause of neonatal cholestasis, as thyroid hormone and cortisol are implicated in bile salts synthesis, transport, and bile acid‐independent bile flow. Unlike primary adrenal insufficiency, electrolyte derangement is less common in ACTH deficiency owing to an intact renin‐angiotensin‐aldosterone system. If present, isolated hyponatremia without hyperkalemia is the most common pattern. In the reported case, the infant presented with conjugated hyperbilirubinemia manifesting initially with clay‐colored stools, which resolved over time with hormonal replacement therapy.

Other than hypoglycaemia, hyponatremia, and cholestasis, neonatal manifestations of SOD include microorchidism/micropenis in male infants, nystagmus, and distinctive facial features involving the midface structures (e.g., frontal bossing, hypertelorism, depressed nasal bridge, long philtrum) [19, 20]. In our case, no apparent dysmorphic features were noted, hence highlighting the heterogeneity of presentation that can be seen in babies with SOD.

Antenatal diagnosis of SOD is particularly challenging because of its subtle prenatal manifestations. With advancements in prenatal imaging, antenatal detection of SOD has improved. Mid‐trimester prenatal ultrasounds may pick up the absence of septum pellucidum from gestational week 20 onwards [21], and this finding may be associated with abnormal development of other brain structures such as optic nerve, or abnormalities/absence of the corpus callosum, which could also serve as the first clue to the prenatal diagnosis of SOD [21, 22]. When prenatal ultrasound findings suggest potential SOD, further imaging with fetal MRI is recommended for detailed visualization of the optic chiasm, optic nerves, and hypothalamic–pituitary region. MRI is useful to evaluate for cortical abnormalities like schizencephaly and polymicrogyria, together known as SOD plus [23]. In our case, antenatal scans did not detect any fetal anomaly, hence highlighting the limitations of antenatal scans in the prenatal diagnosis of SOD.

SOD is a complex congenital disorder with the exact cause remaining largely unknown. The development of SOD is believed to be contributed by both genetic and environmental elements, failing early forebrain development especially during the weeks 4th to 6th of gestation, which is the critical period for anterior neural plate formation [4, 24]. Genetic abnormalities are rare and noted to be identified only in < 1% of patients with SOD [4], with the majority of known SOD cases being non‐hereditary. However, whilst SOD classically has a low rate of genetic diagnosis, a small cohort study of individuals with SOD picked up a genetic etiology in 50% of the families, suggesting that investigation for a genetic etiology is warranted and the incidence of a genetic cause for SOD may be underestimated in the existing literature [9].

Recent breakthroughs in next‐generation sequencing technology have revolutionized variant identification for congenital hypopituitarism [25]. Advances in knowledge of pituitary and forebrain development have led to the identification of critical genes, such as *HESX1, OTX2, SOX2*, and *SOX3*, which are associated with SOD pathogenesis [9, 26, 27, 28]. These genes can be inherited as autosomal dominant or autosomal recessive patterns [28]. A case report showed a Hispanic family with Trio exome sequencing identifying a de novo variant in *SHH*, *NM_000193.2:c.562*+*1G*>*A* in an affected child with clinical SOD, highlighting that there may be an association between *SHH* variants and SOD [9]. There continues to be ongoing research in the genetics association with SOD; with the identification of newer associated genes [9], hence the true epidemiology of SOD with underlying genetic etiology may continue to evolve in the future. Given the recent advances in genetic research, there is good potential for genetic contributions to the diagnosis of SOD in the future. Unfortunately, genetic testing was not done in this infant due to financial concerns.

Environmental factors such as younger maternal age [3, 29], exposure to toxins [30], and vascular or degenerative damage [31] have been considered in existing case series and case reports. Intriguingly, cases without a genetic diagnosis were more epidemiologically associated with being born to younger, primigravida mothers [9]. Our patient's mother was 20 years old, falling in line with the epidemiological risk factors for SOD.

Management of SOD requires multidisciplinary coordination between various healthcare professionals, such as the neonatologist, pediatric endocrinologist, ophthalmologist, and development therapists, to provide the best comprehensive and individualized care [14]. Hormone replacement therapies are needed to address deficiencies and support normal growth and metabolic function [32]. Early ophthalmologic interventions like vision therapy are crucial to optimize the child's visual development [14]. Regular follow‐up and monitoring are essential to constantly evaluate the evolving needs of patients with SOD so that timely interventions can be provided.

## 6 Strengths and Limitations

The robust hypoglycaemia screening program for SGA infants in our center picked up this infant with recurrent hypoglycaemia. Critical blood done for a workup of hyperinsulinism led to the identification of the deficiency of counter‐regulatory hormones, and brain imaging concluded a timely diagnosis of SOD as early as day 13 of life. Early diagnosis and treatment initiation are paramount in reducing long‐term morbidity.

One limitation is that we couldn't manage to get genetic testing for SOD, which would have contributed to the evolving pool of genetic data for patients with SOD. It is worthwhile to consider approaching parents to rediscuss genetic testing again in the future. This is also a single case report, which limits the generalizability of our findings in SOD patients.

## 7 Conclusion

SOD may present in the neonatal period with subtle symptoms and signs of pituitary hormone deficiency, including recurrent hypoglycaemia, conjugated hyperbilirubinemia, and hypothermia. Critical blood sampling in response to recurrent hypoglycaemia is key to picking up any pituitary hormone deficiency, which is essential in guiding the decision for neuroimaging to clinch an early diagnosis of SOD. This was followed up with early imaging of the brain, which led to an early diagnosis of SOD. Early hormone replacement facilitated good growth and developmental outcomes. This case highlights the importance of having a robust hypoglycaemia screening program for infants at risk of hypoglycaemia and the consideration of performing critical blood sampling in infants with recurrent hypoglycaemia, as it may facilitate early detection of rare underlying conditions like SOD.

## Author Contributions

**Yuan Rui Leon Tan:** conceptualization, data curation, formal analysis, investigation, methodology, resources, writing – original draft, writing – review and editing. **Xiaoao Dong:** conceptualization, formal analysis, resources, writing – original draft, writing – review and editing. **Ngee Lek:** conceptualization, writing – review and editing. **Suresh Chandran:** conceptualization, supervision, writing – review and editing. **Odattil Geetha:** conceptualization, supervision, writing – review and editing.

## Funding

The authors have nothing to report.

## Ethics Statement

Case reports are exempted from obtaining ethics approval by Internal Review Board, SingHealth, Singapore.

## Consent

Written informed consent was obtained from the patient to publish this report in accordance with the journal's patient consent policy.

## Conflicts of Interest

The authors declare no conflicts of interest.

## Data Availability

The data that support the findings of this study are available from the corresponding author upon reasonable request.
